# Supplementary material for: Pertussis Toxin Inhibits Encephalitogenic T-Cell Infiltration and Promotes a B-Cell-Driven Disease during Th17-EAE
Source: Int J Mol Sci. 2021 Mar 13;22(6):2924. doi: 10.3390/ijms22062924 (PMC7998427; doi:10.3390/ijms22062924)
Supplement: Supplementary file 1 [file ijms-22-02924-s001.pdf]

Supplement Figure 1

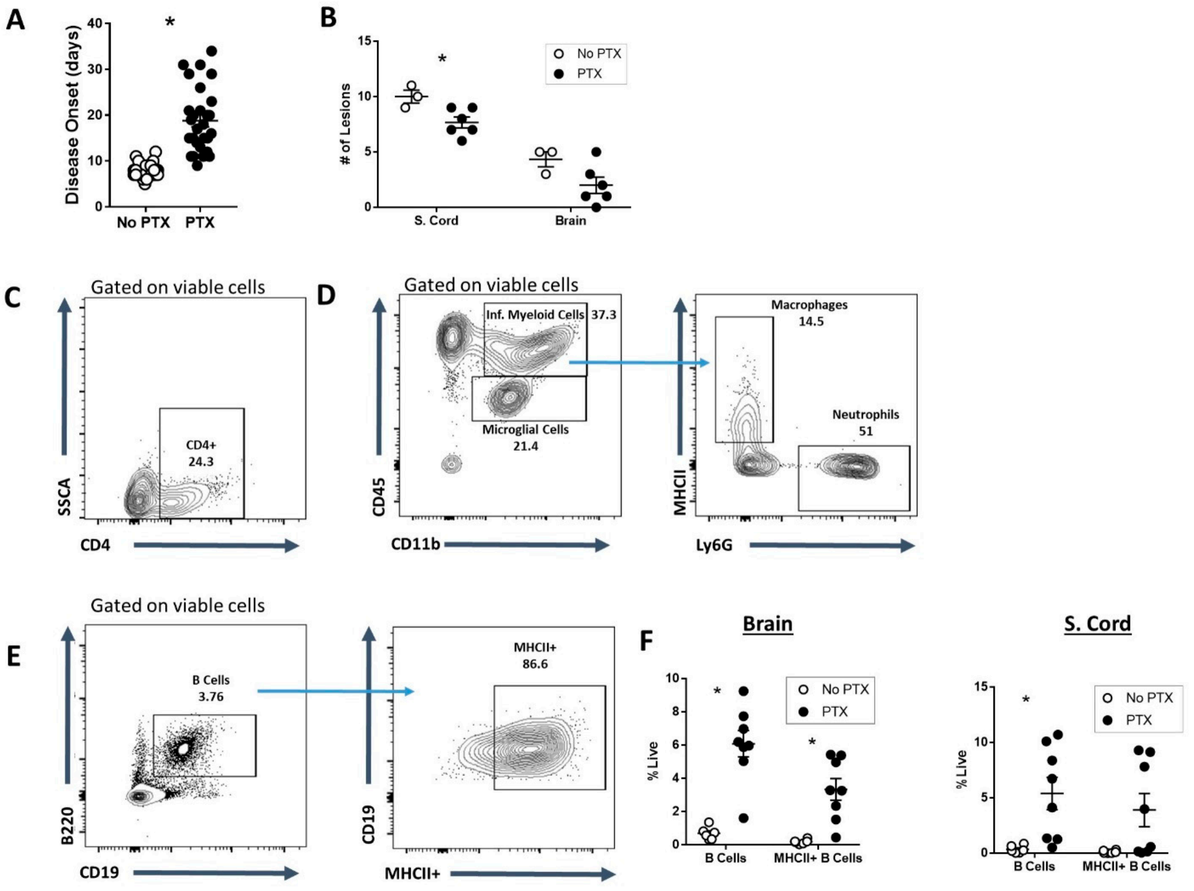

Figure S1:

(**A**) Disease onset of mice treated with PTX or PBS (No PTX); n = 30 mice per group and Mann-Whitney tests were performed to determine statistical significance (*$p < 0.05$). Results compiled from 5 independent experiments. (**B**) Number of demyelinated lesions in the brain and spinal cord of WT Th17-EAE mice treated with (PTX) or PBS (No PTX) at day 0 and 2 (250 ng, IP); n = 3–6/group, compiled from 2 independent experiments. Statistical analysis was performed using Mann-Whitney tests (*$p < 0.05$). Lesions were counted from brain and spinal cord sections, at the peak of disease, stained with H&E and Luxol fast blue. (**C**) Gating strategy for CD4^{+} T cells (**D**) Gating strategy for Microglial cells (CD45^{int}CDllb^{int}), Infiltrating myeloid cells (CD45^{hi}CDllb^{hi}) Macrophages (CD45^{hi}CDllb^{hi}MHCII^{+}ly6G^{-}) and Neutrophils (CD45^{hi}CDllb^{hi}MHCII^{-}ly6G^{+}) (**E**) Gating strategy for B cells (B220^{+}CD19^{+}) (**F**) Frequency of viable B cells and MHCII+ B cells in the spinal cord of mice treated with (PTX) or without PTX (No PTX); n = 6-8/group, student's t-tests were performed to determine statistical significance (*$p < 0.05$). Results compiled from 2 independent experiments.

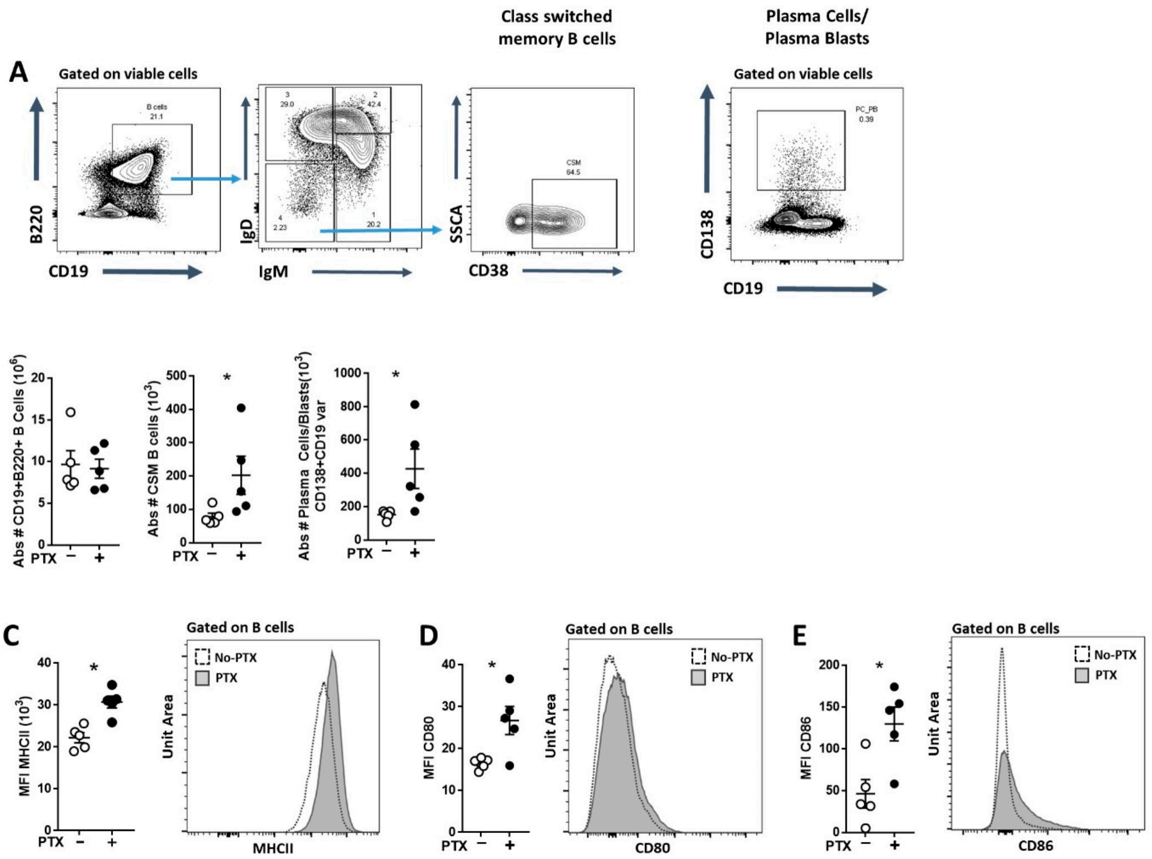

Figure S2:

(**A**) Gating strategy for B cells (B220$^+$CD19$^+$), Class switched memory B cells (B220$^+$CD19$^+$IgD$^-$IgM$^-$CD38$^+$) and plasma cell/plasma blasts (CD138$^+$CD19^{var}) in the spleen of Th17-EAE mice (**B**) Absolute number B cells, class switched memory B cells and plasma cell/plasma blasts in the spleens of WT Th17-EAE mice treated with (PTX) or PBS (No PTX) on day 0 and 2 (250 ng, IP) post transfer; n = 5/group. Statistical analysis was performed using Mann-Whitney tests. (*p < 0.05). Mean fluorescence intensity (MFI) of (**C**) MHCII$^+$ (**D**) CD80 and (**E**) CD86 of B cells in the spleens of WT Th17-EAE mice treated with (PTX) or PBS (No PTX); n = 5/group. Statistical analysis was performed using Mann-Whitney tests. (*p < 0.05).

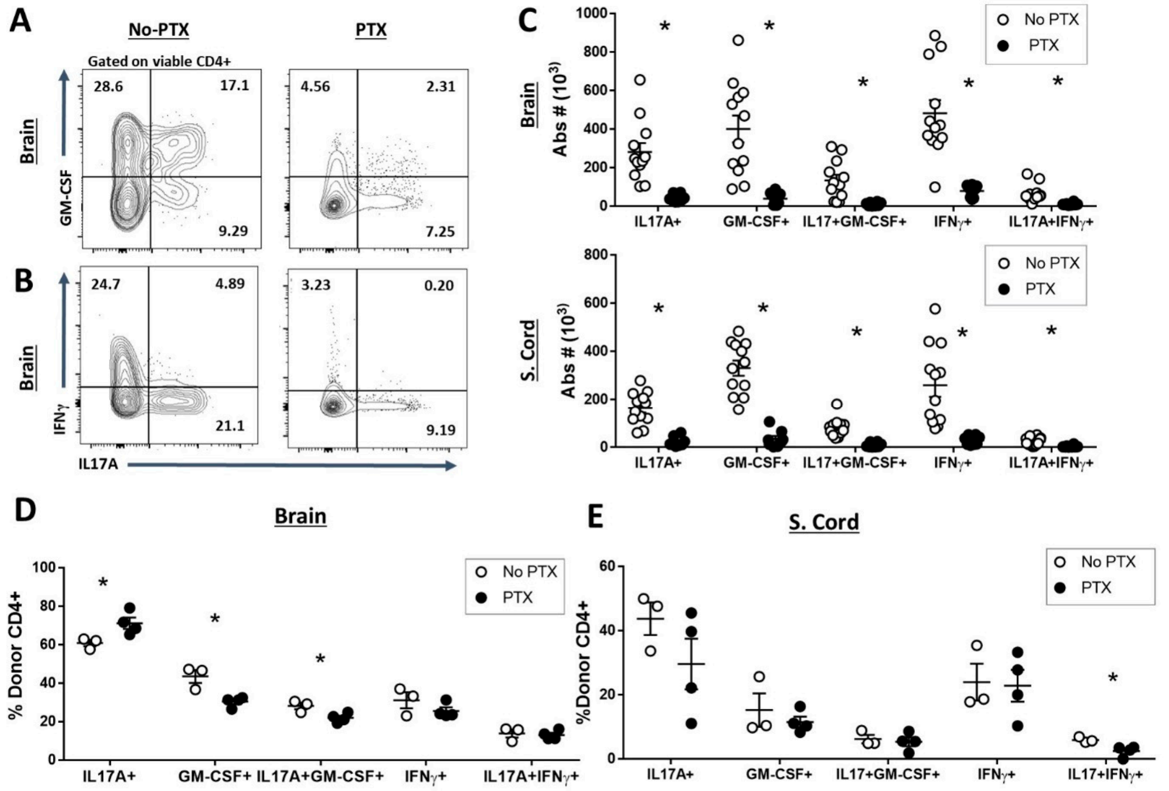

Figure S3:

(A) Representative flow cytometry plots of $CD4^+$ T cells expressing IL17A, GM-CSF or both in the brain of WT Th17-EAE mice treated with (PTX) or PBS (No PTX). (**B**) Representative flow cytometry plots of $CD4^+$ T cells expressing IL17A, IFNγ or both in the brain of WT Th17-EAE mice treated with (PTX) or PBS (No PTX). (**C**) Absolute number of $IL17A^+$, GM-CSF^+, $IFN\gamma^+$, $IL17A^+$GM-CSF^+ and $IL17A^+IFN\gamma^+$ $CD4^+$ T cells in the brain and spinal cord of mice with Th17-EAE treated with (PTX) or PBS (No PTX); n = 12/group, data compiled from 3 independent experiments. Statistical analysis were performed using student's *t-test*. (*$p < 0.05$). Frequency of $IL17A^+$, GM-CSF^+, $IFN\gamma^+$, $IL17A^+$GM-CSF^+ and $IL17A^+IFN\gamma^+$ producing $CD4^+$ cells amongst all $CD4^+$ Donor T cells in the (**D**) Brain and (**E**) Spinal cord of recipient mice with Th17-EAE treated with (PTX) or PBS (No PTX); n = 3-4/group, data compiled from 1 independent experiments. Statistical analysis were performed using student's *t-test*. (*$p < 0.05$).

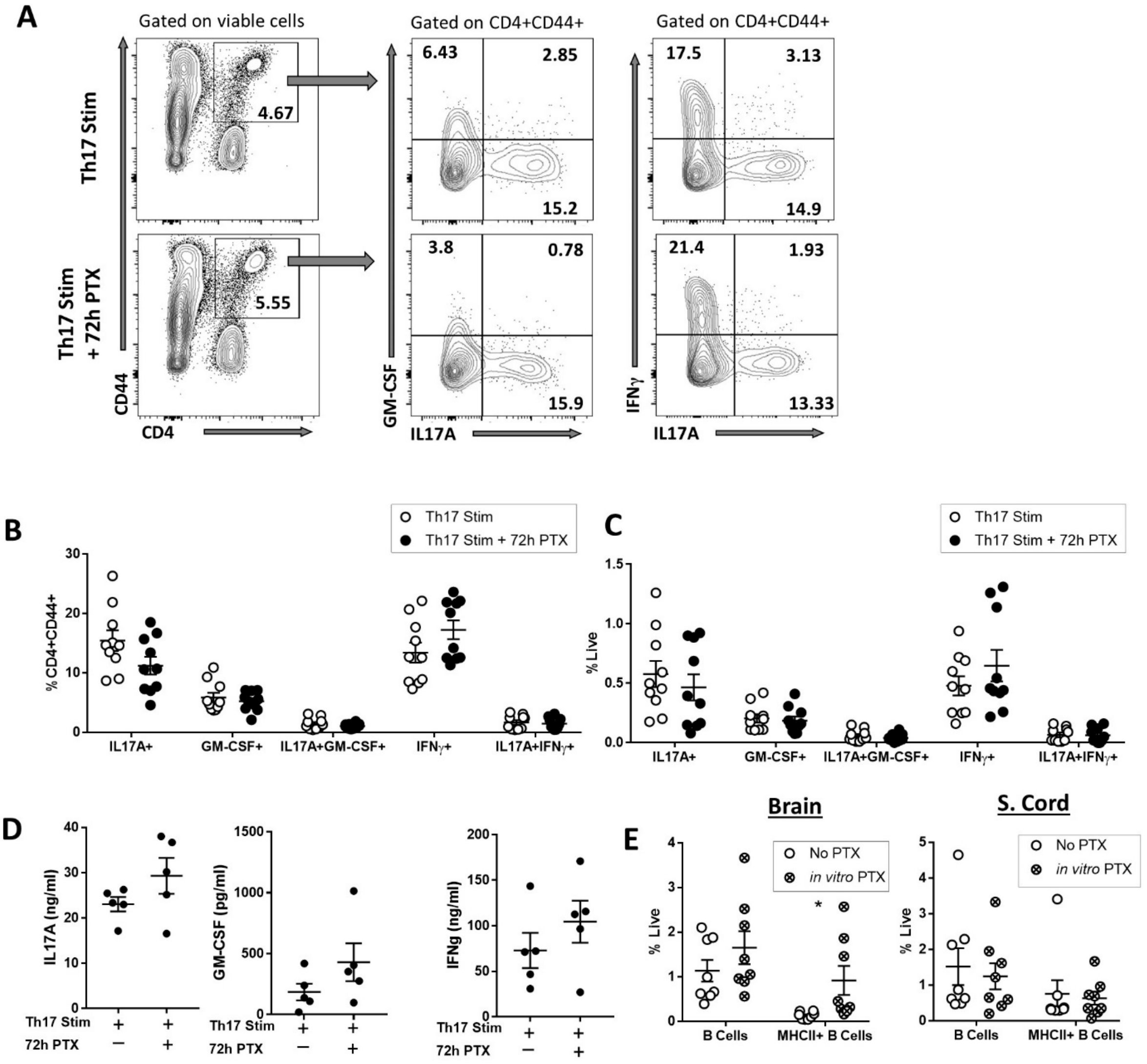

Figure S4:

(**A**) Gating strategy of $CD4^+CD44^+$ T cells and $IL17A^+$, GM-CSF^+, IFNγ^+, $IL17A^+$GM-CSF^+, $IL17A^+$IFNγ^+ expressing $CD4^+CD44^+$ T cells in the post cultured donor cells. (**B**) Frequency of viable IL17A, GM-CSF and IFNγ expressing cells within the $CD4^+CD44^+$ T cell population in the donor cells that were transferred into a recipient mice; n = 5/group. (**C**) Frequency of viable IL17A, GM-CSF and IFNγ expressing $CD4^+CD44^+$ T cells in the donor cells transferred into a recipient mice; n = 5/group. **(D)** The concentration of IL17A, GM-CSF and IFNγ in the Th17 post culture media; n = 5/group. **(E)** Frequency of viable B cells ($CD19^+$) and $MHCII^+$ B Cells in the brain and spinal cord of mice receiving polarized Th17 cells cultured in the presence (in vitro PTX day 27) or absence of (No PTX day 17) of PTX.

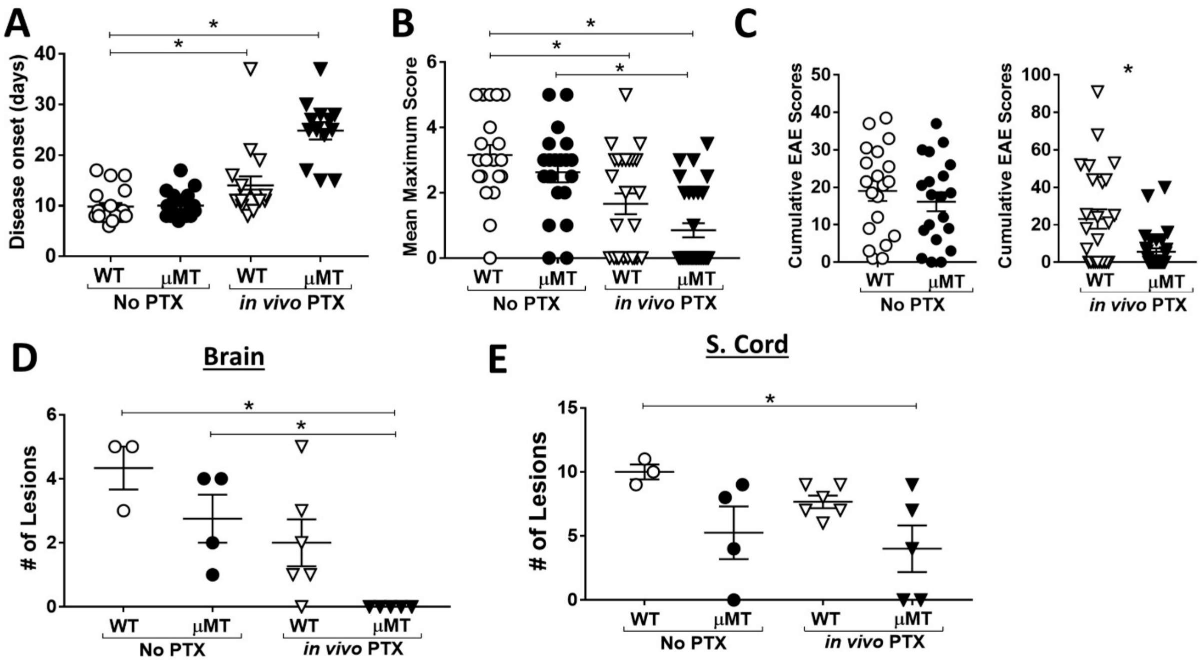

Figure S5:

(**A**) Disease onset (**B**) Mean maximum score and (**C**) Cumulative disease scores (30 days) of WT and μMT Th17-EAE mice treated with PTX (in vivo PTX) or PBS (No PTX) on day 0 and 2 post transfer; n = 25–30/group, data compiled from 5 independent experiments. 1-way ANOVA or Mann-Whitney tests were performed to determine statistical significance (*$p < 0.05$). Number of lesions in the (D) Brain and (E) Spinal cord of WT and μMT Th17-EAE mice treated with or without PTX treatment; n = 3–6/group, compiled from 2 independent experiments. 1-way ANOVA was performed to determine statistical significance (*$p < 0.05$). Lesions were counted from brain and spinal cord sections at the peak of disease stained with H&E and Luxol fast blue.

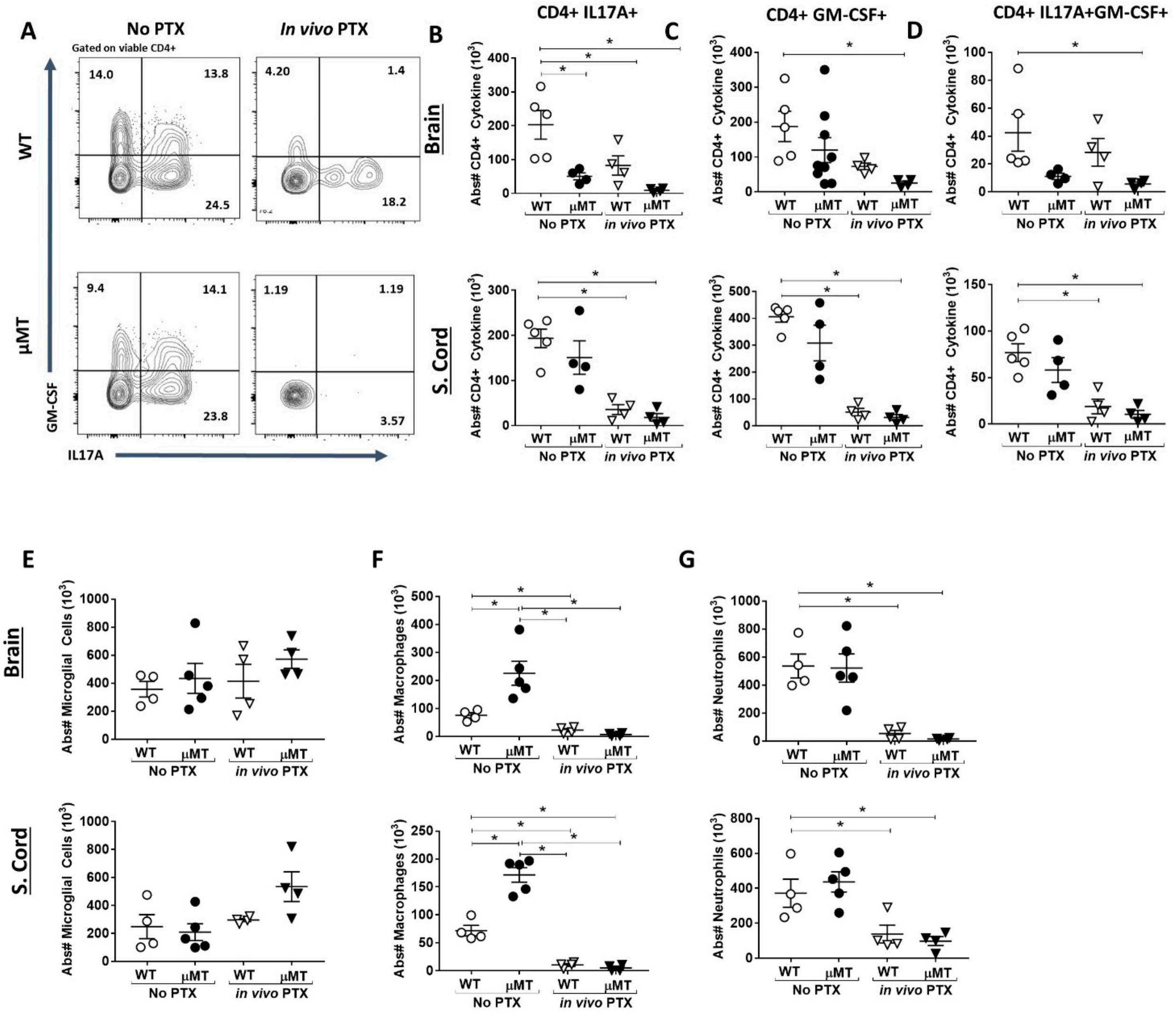

Figure S6:

(**A**) Representative flow cytometry plots of viable $CD4^+$ cells expressing IL17A and GM-CSF in the brain of WT and μMT Th17-EAE mice treated with PTX (in vivo PTX) or PBS (No PTX) on day 0 and 2 post transfer Absolute numbers of viable $CD4^+$ T cells expressing either (**B**) IL17A, (**C**) GM-CSF or (**D**) both IL17A and GM-CSF in the brain and spinal cord; *n* = 4/group, data representative of 2 independent experiments. Absolute number of (**E**) Microglial cells ($CD45^{int}CDllb^{int}$), (**F**) Macrophages ($CD45^{hi}CDllb^{hi}MHCII^{+}ly6G^{-}$) and (**G**) Neutrophils ($CD45^{hi}CDllb^{hi}MHCII^{-}ly6G^{+}$) in the brain and spinal cord; *n* = 4/group. Statistical analysis was performed using student's *t-test* (*$p < 0.05$).

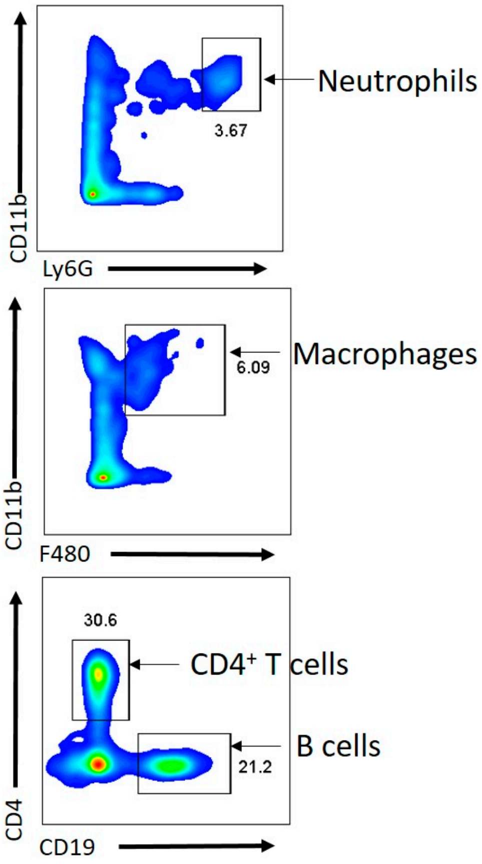

Figure S7:

Gating strategy for B cells ($CD19^{+}$), T cells ($CD4^{+}$), Macrophages ($CDllb^{+}F4/80^{+}$) and Neutrophils ($CD11b^{+}Ly6G^{+}$) corresponding to Figure 6.
